# Phylogenetic characterisation of naturally occurring feline immunodeficiency virus in the United Kingdom

**DOI:** 10.1016/j.vetmic.2011.01.027

**Published:** 2011-06-02

**Authors:** A. Samman, E.L. McMonagle, N. Logan, B.J. Willett, R. Biek, M.J. Hosie

**Affiliations:** a MRC - University of Glasgow Centre for Virus Research, Institute of Infection, Immunity and Inflammation, College of Medical, Veterinary and Life Sciences, University of Glasgow, Bearsden, Glasgow G61 1QH, United Kingdom; b Boyd Orr Centre for Population and Ecosystem Health, College of Medical, Veterinary and Life Sciences, University of Glasgow, Bearsden, Glasgow G61 1QH, United Kingdom

**Keywords:** Feline immunodeficiency virus (FIV), Vaccine, Phylogeny

## Abstract

Feline immunodeficiency virus (FIV) is a significant pathogen of domestic and non-domestic felids worldwide. In domestic cats, FIV is classified into five distinct subtypes (A–E) with subtypes A and B distributed most widely. However, little is known about the degree of intrasubtype viral diversity and this may prove critical in determining whether monovalent vaccines are likely to protect against FIV strains within a single subtype. Here, we characterise novel *env* sequences from 47 FIV strains recovered from infected cats in the United Kingdom and its environs. Phylogenetic analyses revealed that all bar one sequence belonged to subtype A, the predominant subtype in Western Europe. A single sequence was identified as a likely subtype A/C recombinant, intriguing given that subtype C does not appear to exist in either the UK or North Western Europe and suggestive of a recombination event predating its introduction into the UK. Subtype A strains from the UK were not significantly differentiated from representative subtype A isolates found elsewhere suggesting multiple introductions of FIV into the country. Divergence among isolates was comparable to that observed for subtype A isolates worldwide, indicating that FIV in the UK covers the full spectrum of subtype A diversity seen globally. This study demonstrates that while subtype A is predominant in the UK, novel introductions may result in the emergence of novel subtypes or intersubtype recombinants, potentially circumventing vaccine strategies. However, the dominance of subtype A suggests that the development of a regional or subtype-specific protective vaccine for the UK could be achievable.

## 1 Introduction

Feline immunodeficiency virus (FIV) is a widespread pathogen of the domestic cat and infection results in a progressive immune dysfunction similar to AIDS in human immunodeficiency virus (HIV) infection. Since its discovery in 1986 from a cat with an immunodeficiency like syndrome (Pedersen et al., 1987), FIV has been recognized widely as the feline equivalent of human immunodeficiency virus (HIV) and both viruses share significant physical, biochemical and pathogenic features (Johnson et al., 1994). Such are the similarities between FIV and HIV that FIV serves as a valuable animal model for both prophylactic and therapeutic studies of HIV (Elder and Phillips, 1995) as well as being the only non-primate lentivirus that induces an AIDS-like syndrome in its natural host (reviewed in Willett et al., 1997).

Like other retroviruses, FIV has high mutation rate, mainly due to the error-prone reverse transcriptase (Shankarappa et al., 1999); accordingly, diverse viral variants emerge continually in the infected host. Since the *env* gene is the key determinant of viral diversity among FIV (Olmsted et al., 1989), FIV phylogeny studies have focussed mainly on *env* sequences. According to the nucleotide sequence diversity of the V3–V5 region of *env*, FIV is classified currently into five distinct phylogenetic subtypes, designated A to E (Sodora et al., 1994; Pecoraro et al., 1996); subtypes A and B are the most commonly occurring worldwide (Martins et al., 2008). Furthermore, similar to HIV-1, several inter-subtype FIV recombinants have been recognized in natural populations following co-infection; inter-subtype recombinants A/B, A/C and B/D have been identified (Bachmann et al., 1997; Hayward and Rodrigo, 2008).

Phylogenetic studies on FIV sequences have revealed significant heterogeneity (up to 30%) in the sequence of the *env* gene of FIV isolates worldwide (Burkhard and Dean, 2003) similar to that estimated for HIV-1 (Rong et al., 2007). Subtype A isolates are common in Australia, New Zealand, western United States, South Africa and northwestern Europe (Bachmann et al., 1997; Steinrigl and Klein, 2003; Kann et al., 2006a,b, 2007a,b). Subtype B isolates have been identified in the central and eastern United States, central Europe, Brazil and eastern Japan (Sodora et al., 1994; Kakinuma et al., 1995; Pistello et al., 1997; Nishimura et al., 1998; Steinrigl and Klein, 2003; Martins et al., 2008). Subtype C has been recognized in Canada, New Zealand and southeast Asia (Uema et al., 1999; Nakamura et al., 2003; Reggeti and Bienzle, 2004; Hayward et al., 2007). Finally, subtypes D and E are infrequent but were identified originally in southwestern Canada and Japan, and Argentina, respectively (Pecoraro et al., 1996; Nishimura et al., 1998).

Such heterogeneity in *env* gene sequence poses problems for the design of a broadly protective vaccine. It has been reported previously that effective protection was obtained following a homologous FIV challenge using an inactivated whole virus vaccine, although, despite the relative success of Fel-O-Vax against heterologous subtype B isolates (Pu et al., 2005), protection did not extend to a heterologous challenge (Dunham et al., 2006). Such different outcomes highlight the impact of genetic diversity on vaccine strategies against FIV and the importance of assessing the genetic diversity of local subtypes for vaccine development, or before introducing a commercial vaccine to a particular geographical area. Furthermore, identification of the predominant strains in a given region is necessary in order to develop appropriate reagents for the molecular diagnosis of FIV infection (Leutenegger et al., 1999; Hosie et al., 2002).

In order to understand better the degree of intrasubtype viral diversity and the likelihood of a monovalent subtype A FIV vaccine offering broad intrasubtype immunity, we investigated the diversity of FIV within the United Kingdom, a country in which FIV infection is prevalent and where evidence to date suggests that a single viral subtype may dominate.

## 2 Materials and methods

### 2.1 Blood samples, PBMC collection and DNA extraction

Blood samples were collected from 47 FIV sero-positive samples from naturally infected cats submitted to the Companion Animals Diagnostic Services at the University of Glasgow. Cats were either companion pets or had been placed into re-homing shelters. Blood samples were centrifuged at 2000 rpm for 10 min and the plasma removed. Cell pellets were diluted in 1 ml phosphate buffered saline (PBS) and transferred to 20 ml pre-warmed red blood cell lysis buffer (0.88% NH_4_Cl, 10 mM; pH 7.4) and incubated at room temperature for 5 min (red blood cell lysis). The intact white blood cells were then pelleted by centrifugation at 1000 rpm for 5 min. The supernatant was then discarded and the pelleted cells washed twice by centrifugation through ice-cold PBSA buffer (1% bovine serum albumin in PBS). 10^7^ cells were used for DNA extraction using QIAamp DNA Blood Mini Kit (Qiagen, Crawley, UK), as per the manufacturer's protocol.

### 2.2 Cloning of FIV *env* genes and nucleic acid sequence determination

*Env* genes were amplified by using the polymerase chain reaction (PCR) using a high fidelity enzyme mix (Expand High Fidelity PCR system, Roche Diagnostics Ltd., Burgess Hill, UK) and the degenerate primers 1F4 5′-TGTAATCAACG(CT)TTTGT(AG)TC-3′ and 1R4 5′-CCAATA(AC)TCCCAGTCCACCCTT-3′, primers we have found previously to amplify laboratory strains of viral subtypes A, B and C. The FIV env genes were then cloned into the VR1012 eukaryotic expression vector (Vical Inc., San Diego, CA, USA) and their nucleic acid sequences determined using a BigDye Terminator v1.1 kit (Applied Biosystems, Warrington, UK). Sequencing was performed by using Applied Biosystems 3730xl genetic analyser. Raw chromatographic data were analysed by using ‘Contig Express’ sequence analysis software within the Vector NTI suite of programs (Invitrogen Ltd., Paisley, UK).

### 2.3 Multiple sequence alignments and phylogenetic analyses

Nucleotide sequence analysis was performed on a 651 nucleotide fragment spanning the V3–V5 region of *env* gene. The generated consensus included sequences of the 47 isolates included in the study, as well as selected representative sequences from subtypes A–E. Multiple alignment was performed using Clustal X (version 2.0) (Larkin et al., 2007), followed by manual adjustment. Alignments were translated and the resulting amino acid-based alignments used as an exact guide for re-positioning of improper gapping, particularly, where sequences were different in length. Final alignments are available from the authors upon request. DNA distance matrices were calculated with Paup (Swofford, 1993; Wilgenbusch and Swofford, 2003) under a GTR+I+G model, selected in Modeltest (Posada and Crandall, 1998) and parameterized using Maximum Likelihood (ML). A Bayesian phylogeny was estimated in MrBayes under a SDR06 model of evolution (Huelsenbeck and Ronquist, 2001) based on two independent runs of 10 Million generations, with samples taken every 2000 generations. Because initial analyses indicated a problem with inflated branch lengths (Brown et al., 2010), a ML tree (generated in Paup) was added as a starting tree and the branch length prior was adjusted according to the formula provided in (Brown et al., 2010).

Spatial coordinates of the sampled isolates were used to determine their Euclidian distances. In order to test for any spatial patterns consistent with isolation by distance, the correlation between spatial and genetic ML distances was assessed based on a Mantel test using the geodist package in program R (http://cran.r-project.org).

### 2.4 Recombination analysis

A putative recombinant UK sequence (101070) was assessed for evidence of recombination using three different approaches: Bootscan analysis as implemented in the program Simplot, as well as and RDP and GeneConv in program RDP3 (Martin et al., 2010). Along with the 101070 sequence, consensus sequences for each of the five subtypes (A–E) were included in the analysis.

### 2.5 Nucleotide sequence accession numbers

GenBank accession numbers for control reference sequences in the phylogenetic analysis are: Subtype A: X57001 (SwissZ2); M73964 (19K1M); M73965 (19K32); AF531047 (ATVID02); AY221628 (CaONAB02); AY221629 (CaONAB03); AF531033 (CHSHa10); AF531034 (CHTGa05); AF531043 (DEBAb91); AF531037 (DEBAd58); AY196343 (DEBAd59); AY196340 (DENWd60); X60725 (UT113); EF154083 (GBI84); D67052 (JN-BR1); EF154045 (PN13); EF154046 (PN16); AB010401 (PTH-BM3); EF154053 (WST01); EF154076 (WST05); D37813 (Sendai 1); U02404 (USCAlemy_01A); U02411 (USCAtt_01A); U02403 (hnky12); U02410 (sam01); U02413 (tt09); U02417 (zepy01); X69497 (Wales UK14); M36968 (PPR); NC001 (Petaluma); AB010404 (SAP03); L00608 (DIXON); Ca5 (DQ873717); Ca8 (DQ873719); Du1 (DQ873721); Jo5 (DQ873727); Pr3 (DQ873730). Subtype B: AY221627 (CaONAB01); X69501 (M2); X69502 (M3); U02418 (brny03); U02419 (boy03); U02420 (glwd03) U02422 (mtex03); D84497 (Lp9); AB010397 (AICO2). Subtype C: AB016025 (TI1); AB016026 (TI2); AB016027 (TI3); AB016028 (TI4); U02392 (pady02); U02393 (pbar01); U02394 (pbar02); U02395 (pbar03); U02397 (pbar07). Subtype D: AB010400 (OKA01D). Subtype E: D84496 (Lp3); D84498 (Lp20); D84500 (Lp24). GenBank accession numbers for sequences generated in this study are HQ456781–HQ456827 inclusive.

## 3 Results

### 3.1 The sample population

**Fig. 1:**
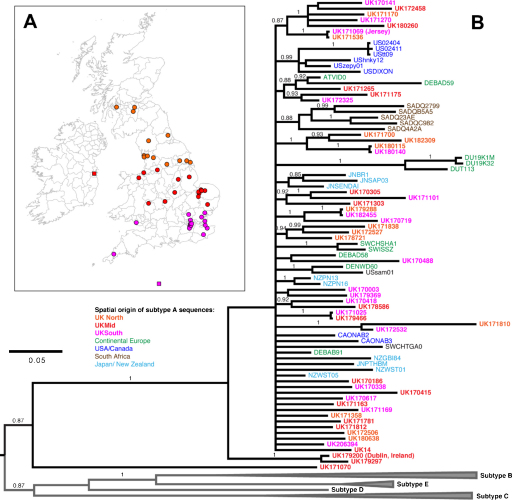
(A) Distribution of FIV isolates included in the study. Map shows the locations of the veterinary practices where blood samples from FIV infected cats were collected. Color-coding distinguishes samples taken from the northern (orange), middle (red), or southern (magenta) part of the sampling area. Red and magenta squares indicate samples from Dublin (Republic of Ireland) and the Isle of Jersey, respectively. Some locations are jittered to enhance their visibility. (B) Bayesian consensus tree of FIV env V3–V5 sequences from the UK (bold font) along with representative sequences of FIV subgroups A–E (clades collapsed). Posterior support values for nodes are shown above branches, values <0.85 are omitted for clarity. Colours for UK sequences as in part A. Other subtype A sequences included were from continental Europe (green), USA and Canada (dark blue), South Africa (brown), Japan and New Zealand (light blue). (For interpretation of the references to color in the figure caption, the reader is referred to the web version of the article.)

Clinical blood samples were examined from 47 animals that had been infected naturally with FIV; the cats were either pet cats or stray cats waiting to be re-homed from animal protection shelters. Samples were collected from across the UK. While the majority of samples originated from England, Scotland or Wales, a single sample came from Jersey (171069) and another from the Republic of Ireland (179200) (Fig. 1). The majority of the cats (35 of 47) were male (74%), a finding that is consistent with previous studies demonstrating the predominant infection of male cats by FIV (Hosie et al., 1989; Reggeti and Bienzle, 2004; Hayward et al., 2007), and likely to be associated with aggressive social behaviour in males increasing the risk of transmission via biting (Natoli et al., 2005).

The ages were known for 34 of the 47 cats and these ranged from 6 months to 16 years, with only 3 cats less than 1 year of age. Infected cats were at different stages of infection, with clinical signs ranging from asymptomatic to symptomatic; clinical signs recorded on the submission forms included lethargy, pyrexia, anaemia, stomatitis, gingivitis, upper respiratory tract infections, urinary tract infections and central nervous system disorders (Table 1).

### 3.2 Phylogenetic analysis of naturally occurring UK isolates of FIV

A Bayesisan phylogenetic tree was generated based on a 651 nucleotide alignment of the V3-V5 region of the *env* gene, comprising 47 novel sequences from naturally occurring isolates of FIV together with reference sequences from subtypes A, B, C, D and E (Fig. 1). The tree demonstrated that all the novel sequences fell within subtype A with 100% posterior support, except for 171070, which did not cluster with any of the known subtypes.

The genetic distance between any two sequences within subtype A ranged between 0.0 and 0.365, with distances among UK isolates (median: 0.110) being virtually identical to those seen among non-UK subtype A isolates worldwide (median: 0.110). These values were lower than the divergence for other subtypes described in previous studies (Sodora et al., 1994; Duarte and Tavares, 2006), indicating that subtype A may be less genetically heterogeneous in comparison with other FIV subtypes.

Although the phylogenetic structure of subtype A sequences was poorly resolved, a number of UK sequences clustered with strong support. This included several pairs (180140/180115; 182455/179288; 171536/171069; 171025/179466) with highly similar V3–V5 sequences, despite having being isolated from geographically distinct locations and regions (Fig. 1). In each case, these sample pairs had been collected and processed on different days; hence sample mix-up or cross-contamination was highly unlikely. A high degree of spatial admixture among UK sequences was also evident from the Mantel test, which failed to reveal a positive correlation between genetic and spatial distances (*r* = −0.010, CI: −0.090 to 0.044; *p* = 0.539).

Notably, British isolates almost never grouped with isolates of other geographic origin; the sole exception being a moderately supported clade that included three UK sequences (171265/171175/172325) and two sequences from continental Europe, DEBAD59 (Germany) and ATVID02 (Austria).

### 3.3 Recombination analysis

**Fig. 2:**
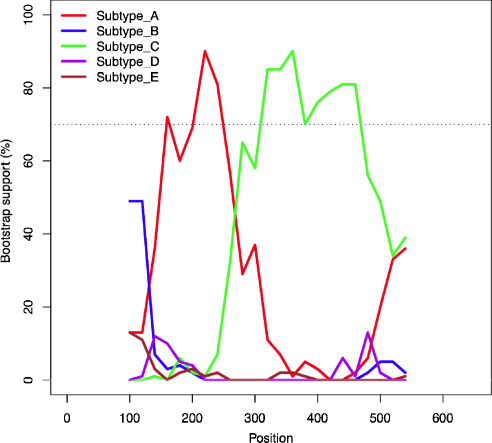
Bootscan analysis of *env* V3–V5 from 171070. Bootscan analysis was performed using a window of 200 nucleotides and a step of 20 nucleotides, under a F84 model with empirical transition/transversion ratio and 1000 bootstrap replicates. Strain 171070 was used as a query in the analysis relative to the subtypes A–E consensus sequences. Horizontal line shows the 70% bootstrap cut-off.

**Fig. 3:**
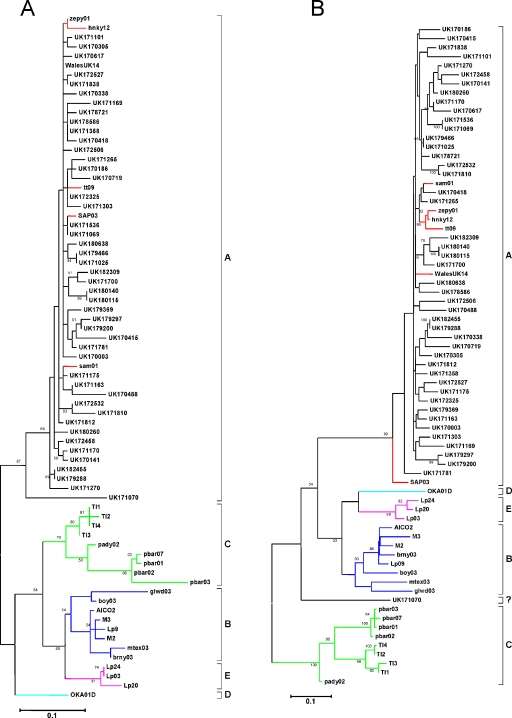
Maximum likelihood phylogenetic trees based on the nucleotide sequence of the A/C recombinant 171070 from (A) 5′ terminus to putative breakpoint sequence, and (B) breakpoint to the end of the sequence. Bootstrap frequencies of 50% or more are shown at the main nodes. Branches are shown in red for subtype A reference sequences, blue for subtype B reference sequences, green for subtype C reference sequences, pink for subtype E reference sequence, and cyan for subtype D reference sequences. (For interpretation of the references to color in this figure legend, the reader is referred to the web version of the article.)

Isolate 171070 was the only British isolate that did not cluster within subtype A and comparisons with sequences representative of subtypes B–E also failed to yield a definitive match. 171070 was isolated from an adopted feral cat and circumstantial evidence leaned towards an introduction to the UK from overseas, possibly from Eastern Europe. Because the virus could not be assigned to any given subtype, we postulated that 171070 may represent a recombinant virus. While further analysis of the sequence using RDP (recombination detection program (Martin et al., 2010) and GeneConv (Sawyer, 1989) found no evidence for a recombination event, bootscan analysis suggested that 171070 may represent a recombinant of subtypes A and C, with a putative breakpoint approximately 260 nucleotides from the start of the *env* sequence (Fig. 2). Phylogenetic trees were constructed based on the criteria of maximum likelihood for the sequences before and after the putative breakpoint. Although the subtype A-like sequence before the putative breakpoint that clustered with subtype A as expected, it failed to fall within the known subtype A. For the subtype C-like portion of the 171070 sequence, assignment was not clear as it did not group with any of the known subtypes (Fig. 3). It is possible that the latter sequence was derived from a subtype C isolate that is distant from reference sequences used to calculate the tree, perhaps derived from Eastern Europe. Unfortunately, reference sequences from this region were not available.

## 4 Discussion

Nucleotide sequences of the V3–V5 region of the *env* gene have been used extensively in the genetic subtyping of FIV strains as well as in molecular epidemiological studies. Although it had been suspected that the FIV isolates circulating within the UK largely belong to subtype A, this is the first phylogenetic study to have been performed in the UK. The results of the current study, based on the sequence of 45 naturally occurring isolates of FIV from across the UK, confirm that subtype A predominates. The diversity of subtype A viruses is thereby remarkably high in the UK, rivaling levels of diversity seen for this subtype at a global scale. This suggests a large number of independent introductions of the virus into the UK. At the same time, phylogenetic resolution of the V3–V5 sequences was relatively poor, precluding insights regarding the possible geographic sources of these introductions as well as introductions into other countries from the UK.

Within the UK itself, the virus population appears to be highly admixed, as no relationship between spatial and genetic distances was apparent from the data. The absence of spatial clustering, likely a consequence of high human mobility, suggests that any new virus subtype being introduced into the UK has the potential to quickly become spatially disseminated. This also includes neighbouring areas, as two of our sequences, originating from Ireland and Jersey, exhibited very limited genetic divergence from strains circulating within the British mainland.

As the only exception to the dominance of subtype A, isolate 171070 was identified as a possible A/C recombinant. This virus was isolated from a feral cat that had been adopted in a suburb near a sea port, raising the possibility that the cat had originated from outside the UK. According to statistics published by the Department for Environment Food and Rural Affairs (DEFRA), 74786 cats entered the UK from 2000 to 2010 under the Pet Travel Scheme (PETS), the system that allows pet dogs, cats and ferrets from a range of European Union (EU) and non-EU countries to enter the UK providing they have been microchipped, vaccinated against rabies and blood tested, and treated for ticks and tapeworms. Cats are not tested for FIV prior to entry into the UK, therefore, animals from areas of the world known to harbour subtype C FIVs, e.g. the USA, may enter the UK provided they have met the requirements for entry under the PETS scheme. The subtype A-like part of the 171070 sequence grouped with, but did not fall within, the known UK viruses (Fig. 3). Therefore, while the specific origin of the putative recombinant is not clear, our results suggest that the recombination event did not involve UK viruses and thus likely took place prior to the virus’ introduction into the country. Since a potential subtype A parent sequence was not found in our data set, it is also unlikely that recombination arose via PCR-mediated recombination. Furthermore, the high fidelity DNA polymerase used to amplify the *env* gene has a low error rate and overcomes many of the limitations of Taq polymerase (Gilje et al., 2008). Interestingly, the position of the putative breakpoint for 171070 (approximately at position 265) was similar to that reported for isolate 214 from New Zealand (Hayward et al., 2007), which could indicate that this site is a recombination hotspot within *env* V3–V5. Hence it appears likely that this is the first isolation of a subtype C-like isolate of FIV from either the UK or Western Europe. Reviewing the NCBI database confirmed that no FIV sequences from Eastern Europe were available for comparison.

Is the predominance of a subtype A in the UK unexpected? It is likely that the dominant subtypes of FIV around the world were established many years ago among geographically isolated populations of cats and may represent multiple introductions of FIV into the cat population. Previous studies have hinted that subtype A viruses may predominate not only in the UK, but in the west of Europe *per se*, in the UK, France, The Netherlands and Switzerland, while subtype B viruses are more common towards the east and south of Europe in Portugal, Italy and Germany (Pistello et al., 1997; Steinrigl and Klein, 2003; Duarte and Tavares, 2006; Steinrigl et al., 2010). Accordingly, the presence of subtype B viruses has been confirmed recently in Turkey (Oguzoglu et al., 2010). Given the physical separation of the UK from mainland Europe, and its nearest neighbors also having subtype A viruses in circulation, the FIV subtypes in the UK cat population may have remained relatively free from the introduction of novel subtypes. The presence of a subtype C recombinant in the UK would suggest a non-European country of origin. The advent of more frequent transport of cats in and out of the UK under the PETS scheme will increase the likelihood of introducing novel subtypes into the UK. Given the prevalence of FIV in the UK is estimated at approximately 5% (Hosie et al., 1989) and the total cat population at approximately 11 million, this would equate to approximately 550,000 FIV-positive cats in the UK. In comparison, in the USA, the prevalence of FIV has been recorded at 2.5% across North America (Levy et al., 2006), while approaching 3.6% in the southern states of Louisiana, Mississippi and Texas (Levy et al., 2007). If we were to consider an extreme scenario whereby all the cats entering the UK were to come from a country such as the USA where subtype C was present, assuming no other biases (for example differences in transmission rates), over a ten year period with 74,786 introductions only 2.5% or approximately 1870 FIV positive cats would enter the UK; 1870 FIV positive cats added to the existing 550,000 subtype A positive cats (0.34%). Thus, *in extremis*, we might expect to detect one novel introduced subtype in approximately every ∼300 cats sampled. Our study examined 47 FIV-positive cats, it is therefore somewhat fortuitous that we detected the novel A/C recombinant. This theoretical consideration does emphasise that it may take a considerable time for the composition of subtypes in the UK to alter.

Since Env, the major target for virus neutralization, displays immense diversity, it has been proposed that it may be necessary to develop regional vaccines that are each based on the sequence of the predominant circulating viruses in particular regions or countries, at least as a preliminary controlling measure (Pistello et al., 1997; Steinrigl and Klein, 2003). There is some evidence that protection can be achieved against an intra-subtype B heterologous FIV challenge using an attenuated FIV vaccine (Pistello et al., 2003). However this trial was conducted solely with FIV isolates from subtype B and this finding has not been reproduced in trials involving other subtypes or in a wider geographical area. It has been suggested that subtype B isolates may be more ancient and host adapted and as a consequence may be less virulent (Bachmann et al., 1997). In countries where a more heterogeneous FIV population exists, an effective vaccine would be required to protect against diverse strains which may belong to more than one subtype. Moreover, in such circumstances, recombination between FIV isolates of different subtypes may occur as a result of co-infection and result in chimaeric strains which may be resistant to vaccine-induced protection. Our results suggest that such a scenario is less likely for the UK, which appears to be strongly dominated by subtype A virus and thus may a suitable area for a subtype A vaccine trial, should such a vaccine become available.

In conclusion, this study has demonstrated that FIV subtype A, if not exclusively the circulating subtype, predominates in the UK. Further characterisation of the genetic diversity of FIV investigating a greater number of isolates may provide more information about whether subtype A is the only subtype circulating within the UK, since an A/C recombinant isolate has been recognised. Because of the immense diversity of the virus, developing regional or sub-type specific vaccine could be of significant use.

## Figures and Tables

**Table 1:** Details and clinical signs of the naturally FIV-infected cats included in the study.

Strain	Location	Age	Gender	Health status	Clinical signs	Subtype
170003	Essex	2–3 years	Male	NK	NK	A
170141	Kent	12 years	Male	NK	NK	A
170186	Norfolk	15 years	Male	Sick	Anaemia	A
170305	Suffolk	NK	Female	NK	NK	A
170338	Essex	11 years	Male	Sick	NK	A
170415	Lincolnshire	Adult	Male	NK	Covered in scabs and scars	A
170418	London	1 year	Male	NK	NK	A
170488	Kent	4 years	Male	NK	Pallid, poor condition	A
170617	London	Adult	Female	Sick	NA	A
170719	London	9 years	Male	Sick	Anaemia – dehydration	A
171025	London	2.5 years	Male	NK	NK	A
171069	Jersey	NK	Male	Healthy	No lesions apparent	A
171070	Worcestershire	8 years	Male	Sick	Ulcerative pharyngitis	A/C
171101	London	6 months	Male	Sick	Alopecia	A
171163	Leicester	9 months	Female	Sick	Cyclical pyrexia	A
171169	Cornwall	1.5 years	Female	Healthy	Slight conjunctivitis	A
171170	Lancashire	Adult	Male	Healthy	No lesions apparent	A
171175	Norfolk	16 years	Female	Sick	Upper respiratory tract signs	A
171265	Nottinghamshire	NK	Male	Healthy	No lesions apparent	A
171270	Cheshire	3 years	Male	Healthy	No lesions apparent	A
171303	Norfolk	Adult	Male	Sick	NK	A
171358	Lanark	16 years	Male	Sick	Persistant non-regenerative anaemia, leucopenia	A
171532	Cumbria	5 years	Male	Sick	Recurrent gingivitis/stomatitis	A
171700	Hull	NK	Male	NK	NK	A
171781	Norfolk	10 years	Male	Sick	Anorexia, recurrent gingivitis	A
171810	Lancashire	NK	Female	Sick	Change of coat color	A
171812	Norfolk	5 years	Male	Healthy	No lesions apparent	A
171838	Glasgow	1.5 years	Female	Sick	Dullness, inappetance	A
172325	London	12 years	Female	NK	NK	A
172458	Wirral	4 years	Male	Sick	Gingivitis – stomatitis	A
172506	Hull	10 years	Male	Sick	NK	A
172527	Glasgow	9 years	Female	Sick	Ataxia	A
172532	Essex	NK	Male	NK	NK	A
178586	Manchester	5 years	Female	Sick	Severe gingivitis	A
178721	Lancashire	4–5 years	Male	Sick	Large abscess on face	A
179200	Dublin/Ireland	10 years	Male	Sick	Weight loss, lethargy, fever	A
179288	Lancashire	3 years	Male	Sick	Mild gingivitis, neck lesions	A
179297	Norfolk	8 years	Male	Healthy	No lesions apparent	A
179369	Hertfordshire	Adult	Male	NK	NK	A
179466	Worcestershire	5 years	Male	NK	NK	A
180115	Dewsbury	12 years	Female	Sick	Mucopurulent ocular and nasal discharges, stomatitis, bilateral conjunctivitis	A
180140	London	Adult	Male	Healthy	No lesions apparent	A
180260	Denbigh	10 years	Male	NK	NK	A
180638	Durham	>2 years	Male	Sick	Severe periodontitis, swollen and lysed jaw bones	A
182309	Hull	9 years	Male	Sick	Inappetance – halitosis	A
182455	Bridgend	NK	Female	NK	NK	A
206394	Kent	11 years	Male	Sick	Urinary tract infection	A

NK, not known.
